# Genes Are Often Sheltered from the Global Histone Hyperacetylation Induced by HDAC Inhibitors

**DOI:** 10.1371/journal.pone.0033453

**Published:** 2012-03-30

**Authors:** John Halsall, Vibhor Gupta, Laura P. O'Neill, Bryan M. Turner, Karl P. Nightingale

**Affiliations:** Chromatin and Gene Expression Group, Institute of Biomedical Research, College of Medical and Dental Sciences, University of Birmingham, Birmingham, United Kingdom; St. Georges University of London, United Kingdom

## Abstract

Histone deacetylase inhibitors (HDACi) are increasingly used as therapeutic agents, but the mechanisms by which they alter cell behaviour remain unclear. Here we use microarray expression analysis to show that only a small proportion of genes (∼9%) have altered transcript levels after treating HL60 cells with different HDACi (valproic acid, Trichostatin A, suberoylanilide hydroxamic acid). Different gene populations respond to each inhibitor, with as many genes down- as up-regulated. Surprisingly, HDACi rarely induced increased histone acetylation at gene promoters, with most genes examined showing minimal change, irrespective of whether genes were up- or down-regulated. Many genes seem to be sheltered from the global histone hyperacetyation induced by HDACi.

## Introduction

The post-translational modification of core histones plays a central role in epigenetic gene regulation [1]. Modifications are put in place by families of modifying and demodifying enzymes, the activities of which are influenced by local concentrations of metabolites or environmental agents [2], providing an interface by which the environment can interact with the genome [3]. Recent studies have begun to define the genomic distribution of specific histone modifications and to link these to gene expression [4]. These approaches reveal associations, such as higher levels of histone acetylation at the promoters of active genes, however, it remains uncertain whether these modifications are a consequence of ongoing processes (i.e. gene activity at adjacent loci), or are predictive or causative of future transcriptional states [5].

Studies on the functional consequences of histone modification(s) frequently use enzyme inhibitors to manipulate the abundance of specific modifications. Salts of short chain fatty acids (e.g. butyric, propionic, acids) occur at millimolar concentrations in the mammalian large intestine, and have been known for many years to induce histone hyper-acetylation in cultured cells [6]. They do this by inhibiting members of the histone deacetylase (HDAC) family, enzymes which together with histone acetyl transferases, maintain the dynamic distribution of histone acetylation across the genome [2]. Valproic acid (VPA) is a branched short-chain fatty acid HDAC inhibitor (HDACi) that is used clinically as a preventive treatment for seizures and bipolar disorder [7]. More recently VPA and other HDACi have been shown to be effective chemotherapeutic agents [8], [9], however it remains unclear how these reagents suppress tumour cell growth. Given the long-standing association between histone acetylation and transcriptional activity [10], the global increases in histone acetylation induced by HDACi might be expected to lead to widespread increases in gene expression. However, analysis indicates that only a small proportion of genes are up-regulated by these agents [11], [12], and whether histone acetylation changes at these loci is controversial. Recent genome wide analysis indicated that HDACi induce histone acetylation at transcriptionally active, but not silenced genes [13], however a comparable study detected only transient increases in acetylation, and prolonged deacetylation at many genes [14]. This, and the recognition that HDACi induce global changes in other histone modifications [15] and impact on the acetylation of non-histone proteins [16], suggest that the mechanisms that underpin gene responses to HDACi are complex [17].

Here, we explore the relationship between the genome-wide histone hyperacetylation and transcriptional responses induced by VPA, and how this relates to histone modification at selected genes. We find this HDACi does not increase histone acetylation at gene promoters and coding regions, even at genes showing enhanced transcription. This indicates that genes are often unaffected by the HDACi-induced genome-wide histone hyperacetylation, and suggests that mechanism(s) other than increased histone acetylation are responsible for the transcriptional responses to this agent.

## Materials and Methods

### Cell culture and cell cycle analysis

Human HL60 (promyelocytic leukaemia) cells were cultured in RPMI 1640 medium supplemented with 8% foetal bovine serum (Invitrogen), 100 µg/ml streptomycin and 100 U/ml penicillin at 37°C, 5% CO_2_. Where required, sodium valproate (5 mM, Sigma), SAHA (2.5 µM, gift of Dr PA Marks, Sloan-Kettering Cancer Center. New York), or TSA (165 nM) was added. For cell cycle analysis cells were washed once in Phosphate Buffered Saline (PBS), fixed in 80% ethanol and resuspended to 10^6^ cells/ml in PBS supplemented with 1 mg/ml RNase A and 0.2 mg/ml propidium iodide. Cells were analysed on a Coulter XL flow cytometer.

### Expression microarrays

Total RNA was isolated from HL60 cells using a Qiagen RNeasy Mini kit, cDNA generated using Superscript III reverse transcriptase (Invitrogen), and purified using a Qiagen PCR purification kit according to the manufacturers' instructions. cDNA quality was checked by PCR amplification of a fragment of β-actin.

cDNA was labelled with Cy3 or Cy5 using a Bioprime labelling kit, and random primers (Invitrogen), purified using the Qiagen PCR purification kit as above. Labelled probes were denatured and hybridized at 42°C for 16–20 hours. Thereafter slides were washed sequentially in 2xSSC 0.1% SDS, 0.2xSSC and 0.05xSSC. Slides were scanned using an Axon Genepix 4000B scanner and read by Genepix 3.0 software.

Initial experiments used HGMP5K cDNA arrays with 11520 elements representing 5000 genes. Three independent experiments were performed. Data was collected from the green (Cy3) channel and quantile normalised across all samples. Student's t-test was used to compare results for VPA-treated and untreated samples for each gene and calculate the significance of any differences (*p* value) and false discovery rate (*q* value) using the R statistical package and Q-Value software. Only genes with a *q* value of less than 0.1 (i.e. a less than 10% chance of being false positives) were taken forward for further analysis. Operon microarrays (Operon Biotechnologies, AL, USA) contain elements representing 25 823 genes. Labelled cDNA from VPA-treated or untreated HL60 cells were hybridised in parallel together with reference cDNA supplied by the manufacturers. Three independent experiments were performed. Raw data was analysed by the GEPAS programme [18] and Loess normalised [19]. Changes in gene expression detected by microarray analysis were validated by RT-qPCR using SYBR Green PCR Mastermix (Applied Biosystems) and appropriate primers (*Supplementary Table S1*.). Expression microarray data are MIAME compliant, and have been deposited with the Geo database (Accession number pending).

### Chromatin Immunoprecipitation (ChIP)

ChIP was performed on 5×10^7^ HL60 cells grown overnight in the presence of 1 µCi/ml ^3^H-thymidine (Amersham). ChIP was carried out as previously described [20]. In short, unfixed cells were digested with micrococcal nuclease to yield mono-nucleosomal fragments, incubated overnight with affinity purified rabbit polyclonal antibodies and the antibody-bound material isolated on protein A-sepharose beads (Invitrogen). DNA from antibody-bound (‘Bound’) and unbound fractions (‘Unbound’) was purified by phenol-chloroform extraction and quantified by qPCR. The relative abundance of a modification (*‘Enrichment’*) from ChIP experiments are calculated as a ratio of the material detected in the *‘Bound’* and *‘Unbound’* fractions (B/UB). As such values above 1.0 indicate that the modification is enriched at the locus detected. Primer pairs used for analysing ChIP DNA are listed in Supplementary Table S2. Rabbit polyclonal antisera with the following specificities were either prepared in house; H4K8ac (R403), H4K16ac (R252), H3K9ac (R607) and H3K4me3 (R183), or purchased (H3K9me2, Millipore). All antibody specificities were confirmed by inhibition ELISA and western blotting.

## Results

### HDACi induce genome-wide histone hyperacetylation

**Figure 1:**
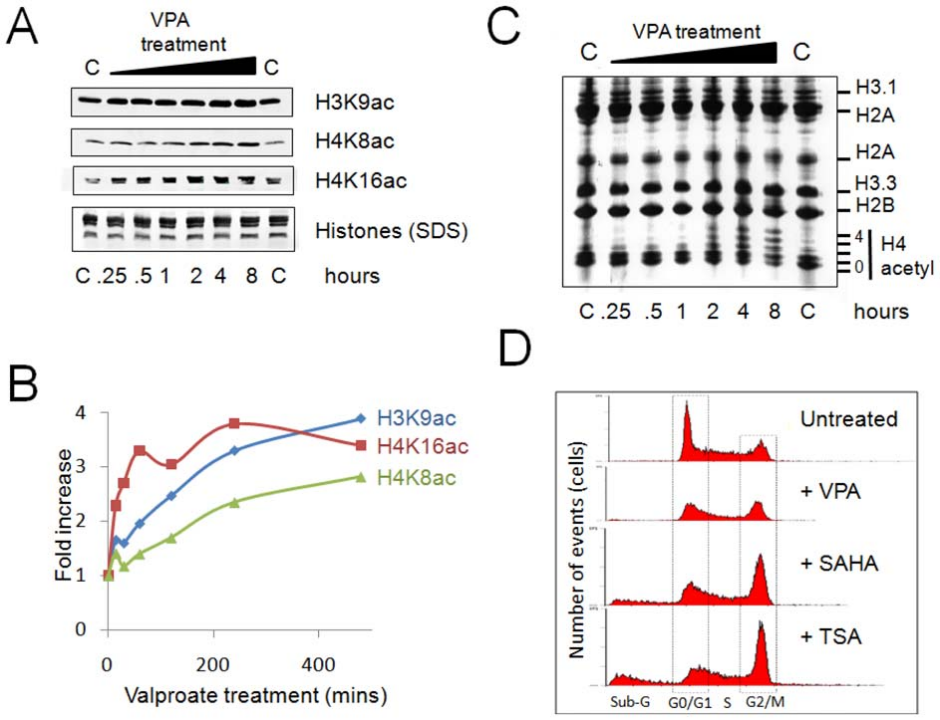
Changes in bulk histone acetylation and cell cycle progression induced by HDACi. (**A**) Western blots, and (**B**) quantification of the changes induced in H3K9ac, H4K8ac, H4K16ac abundance upon VPA treatment (5 mM, 8 hours). (**C**) Triton-acid-urea gel of histones isolated from control (C), or VPA–treated cells over the same time course. Histone H4 associated with none, one or more acetyl residues are indicated. (**D**) *HDACi treatment impacts on the cell cycle.* Fluorescence activated cell sorting of untreated and VPA (5 mM), SAHA (2.5 µM) or TSA (165 nM) treated cells (8 hours). Cells associated with distinct stages of the cell cycle are indicated. Cells labelled Sub-G are either dead or apoptotic.

As a model system for these experiments, we used the human promyeloid leukaemia cell line HL60 [21]. We have shown previously that HL60 cells exposed to HDACi exhibit a rapid increase in histone acetylation, which plateaus after ∼8 hours of treatment [15]. Western blotting with residue-specific antibodies confirmed these data – indicating a rapid response, and similar increases in H3 and H4 acetylation observed after eight hours of HDACi treatment (Fig. 1A,B). Subsequent triton-acid-urea gel analysis confirmed these changes are genome-wide (Fig. 1C), as the bulk of histone H4 is non or mono-acetylated in untreated cells, but ∼80% of histone H4 is acetylated after eight hours of treatment.

### Cell cycle response to HDACi

We characterised the cellular response to three inhibitors at concentrations that induced similar levels of global histone hyperacetylation [22]. These include two clinically important agents, suberoylanilide hydroxamic acid (SAHA) and sodium valproate (VPA), and the research tool, trichostatin A (TSA). Flow cytometric analysis of the DNA content of treated cells indicate that HDACi induce changes in cell cycle progression, but the profiles show subtle differences. All three agents induce an accumulation of cells in G2/M, but this is more pronounced with SAHA and TSA, which also stimulate apoptosis. In contrast, VPA leads to an accumulation of material in G2/M but minimal apoptosis (Fig. 1D).

### Gene expression changes in HDACi-treated cells

**Figure 2:**
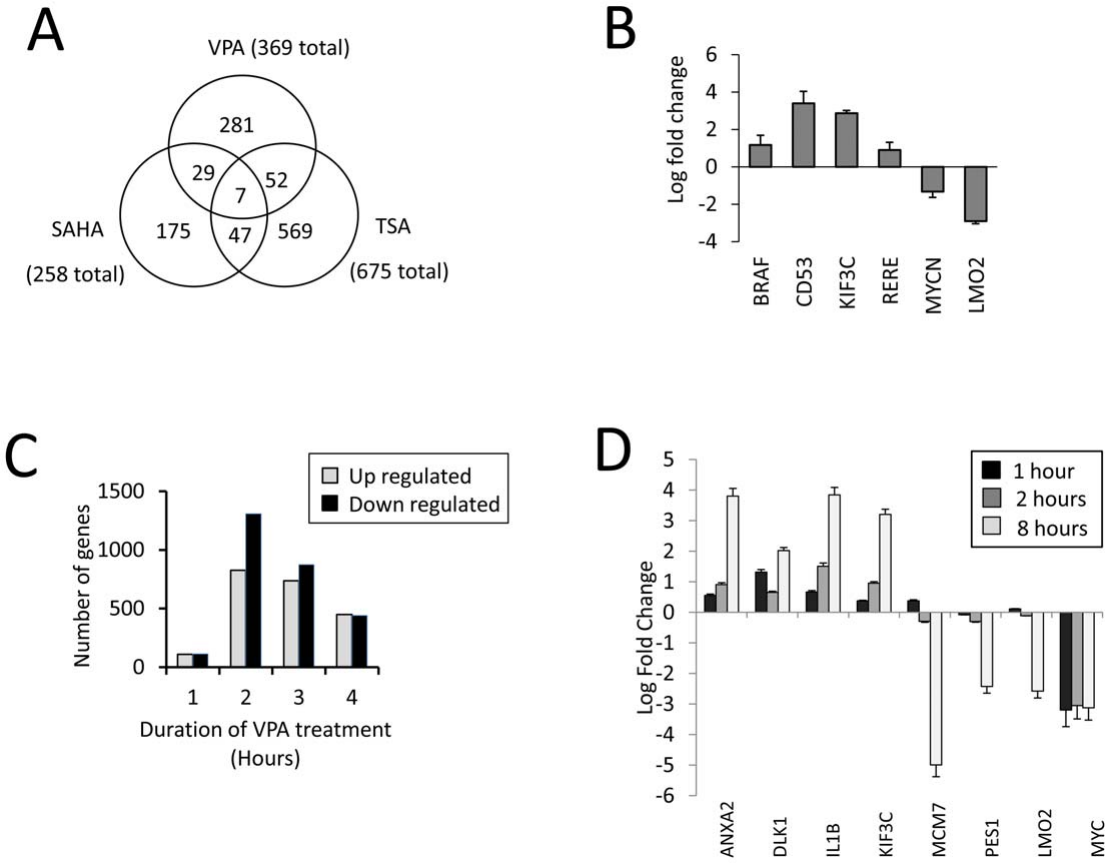
Changes in transcript level induced by HDACi. (**A**) Venn diagram showing genes where transcript levels changed significantly (p<0.01) in response to HDACi, detected by HGMP 5K array (5000 gene probes). HL60 cells were treated with VPA (5 mM), SAHA (2.5 µM) or TSA (165 nM) for eight hours. (**B**) RT-qPCR was used to quantify the transcriptional change at genes that responded to all three HDACi. HL60 cells were treated with VPA (5 mM, 8 hours). Transcript abundance was normalised to β-actin, and presented on a base 2 logarithmic scale. Values are presented with a standard error of mean (*n = 3*). (**C**) Cells were treated with VPA (5 mM), and transcript abundance analysed at defined time points using *Operon* microarrays (25823 genes). Quantitative presentation of the number of genes that were more than 2-fold up-regulated (*Shaded bars*) or down-regulated (*Filled bars*) at time points during the time course. (**D**) RT-qPCR was used to quantify the transcriptional change at genes that responded to VPA (*5 mM*) at 1, 2 and 8 hours treatment. Genes show divergent responses, including early and continued up-regulation (*ANXA2, DLK1, IL1B, KIF3C*), little initial change and subsequent down-regulation (*MCM7, PES1, LMO2*) or early and persistent down-regulation (*MYC*). Transcript abundance was normalised to β-actin, and presented on a base 2 logarithmic scale.

As an initial screen to identify genes in HL60 cells whose expression changes in response to HDACi, we exposed log-phase cells to the same inhibitor treatment regimens. Cells were treated for eight hours and cDNA from treated and untreated cells hybridized to HGMP5k cDNA arrays. The labelling intensities of probes hybridized with cDNA from treated and untreated samples were compared (t-test) and the significance of the difference (*p*-value) calculated (*See Methods for details of data processing*). Analysis of the responding genes showed a number of unexpected findings (Fig. 2A). Of ∼5000 genes on the slide, only 369 (∼7%) showed a significant (*p*<0.01) change in response to VPA, of which broadly equivalent numbers of genes were down-regulated (154 genes) as up-regulated (215 genes). Similar responses were also induced by the hydroxamic acid-based HDACi, TSA and SAHA. Both agents induced transcriptional change at a small proportion of genes (SAHA, 258 genes (5%); TSA: 675 genes (13%)), with broadly equivalent numbers being down- and up-regulated. Although all three agents induced similar patterns of response, this involved distinct populations of genes (Fig. 2A), even with TSA and SAHA which share a common structural identity. Importantly, only seven of 1167 responding genes respond to all three agents, despite all inhibitors inducing similar degrees of histone hyperacetylation. These results were validated by re-analysing selected genes that showed a maximal response to VPA treatment (within the top 10%). Real time PCR analysis (Fig. 2B) confirmed both the direction, and magnitude of the transcriptional response at these loci.

### Identifying genes showing early VPA-induced changes in transcription

Our subsequent studies focussed on identifying VPA-responding genes at early time points, as we reasoned that these are more likely to be induced by the direct action of the inhibitor. To do this, we used a larger array (25 823 genes), allowing us to identify the few genes that showed changes in transcript abundance (>2-fold) after only one hour of treatment (*223 genes, 0.8%*). The maximal transcriptional response was at two hours (*2134 genes*, Fig. 2C). These data are consistent with the earlier study, in that only a small proportion of genes respond to VPA (∼9%, *2 hours*), and that as many genes are down-regulated as up-regulated, even after short exposure times (*1 hour; 110 up-regulated, 113 down-regulated*).

These data were validated by RT-qPCR, focussing on genes that showed early increases in expression, or were down-regulated after protracted VPA treatment. Transcriptional activity was probed at several time points through the VPA treatment to assess the complexity of transcriptional responses (Fig. 2D). Interestingly the majority of genes showed progressive changes in expression – with both up-regulated (*ANXA2, DLK1, ILB, KIF3C*), and down-regulated genes (*PES1, MYC*) showing consistent behaviour throughout the timecourse. For two genes (*MCM7, LMO2*), an initial (small) up-regulation (1 h) was followed by much more pronounced down-regulation (8 hr).

### Gene-specific changes in histone modification

**Figure 3:**
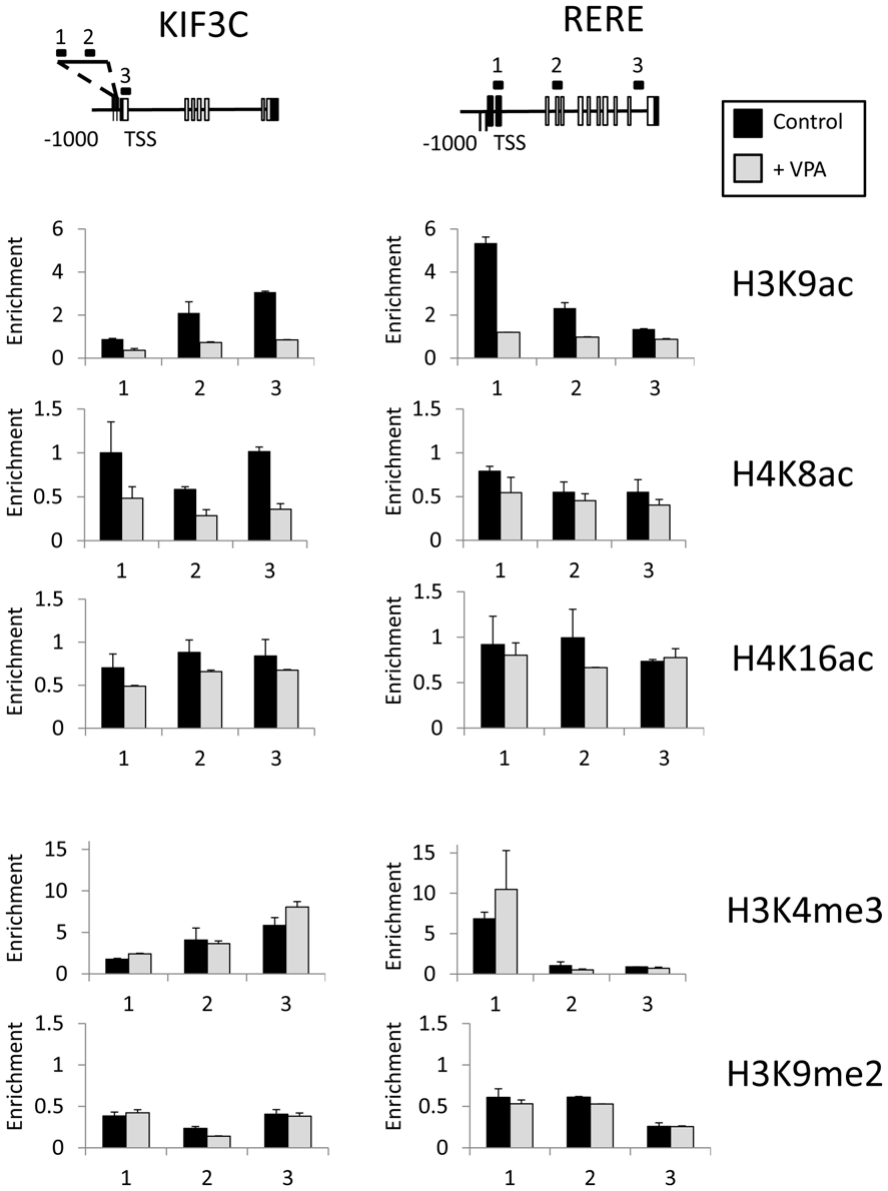
Histone modification distribution on genes up-regulated by VPA. Chromatin immunoprecipitation analysis of histone modifications on two VPA-responsive genes: *KIF3C* and *RERE*. Graphs indicate the enrichment of histone modifications at specific primer pairs in untreated cells **(Control),** and after eight hours of VPA treatment **(+VPA).** Histone modification enrichment is shown as a bound∶unbound ratio. The primers used are indicated on gene diagrams, which also indicate the transcription start site (TSS), exons (Empty boxes), and untranslated regions (Filled boxes). The abundance of a histone modification (‘*Enrichment*’) is calculated from the ratio of material detected in bound∶unbound fractions.

The expression analyses described indicate that VPA stimulates transcriptional responses at a subset of genes. We used chromatin immunoprecipitation to assess whether transcriptional activation correlated with increased histone acetylation at two VPA up-regulated genes - *KIF3C* (*7-fold increase*) and *RERE* (*2-fold increase*) (Fig. 3). Epigenome mapping indicates that the TSS of active genes show enhanced levels of acetylated histones [13], so we focussed on these regions. Acetyl marks are not functionally interchangeable, so we analysed a highly abundant acetyl mark (H4K16ac), along with marks found in moderately acetylated (H4K8ac) or hyperacetylated chromatin (H3K9ac) [23]–[25].

This analysis showed acetylation distributions on these genes in untreated cells are as expected. H3K9ac, a mark associated with hyperacetylated chromatin, showed significant enrichment at the TSS (*Primer 3, KIF3C; Primer 1, RERE*), but not at sites either upstream or within the body of the genes. In contrast, the more abundant acetyl marks, H4K8ac and H4K16ac, were not enriched within these genes. These data are consistent with previous studies [13]. However the distribution of acetyl marks after VPA treatment was surprising. We predicted that levels of acetylation would increase, given the increased gene activity at these two genes. However, our data did not show this – detecting either pronounced (H3K9ac, H4K8ac), or modest (H4K16ac) falls at the sites analysed. These changes were most significant at promoter-proximal regions, where acetylation levels were relatively high in untreated cells. In contrast, other histone marks act as expected. H3K4me3, a mark associated with transcriptional activation, is highly enriched on both gene promoters in untreated cells, and shows a modest increase upon VPA treatment (Fig. 3). Similarly, both genes are associated with low levels of the ‘repressive’ mark H3K9me2, which did not significantly change upon VPA treatment (Fig. 3).

Levels of H3K8ac and H4K16ac were also assessed on the VPA-responsive genes, *CD53*, (*11-fold increase*) and *BRAF* (*2-fold increase*) and also showed minimal changes in histone acetylation upon VPA treatment (*Data not shown*). Thus, for all four genes, there is a mismatch between the genome-wide hyperacetylation induced by VPA and the stability, or reduction of histone acetylation at promoter regions.

### Time course of histone modification changes on transcriptionally up-regulated genes

**Figure 4:**
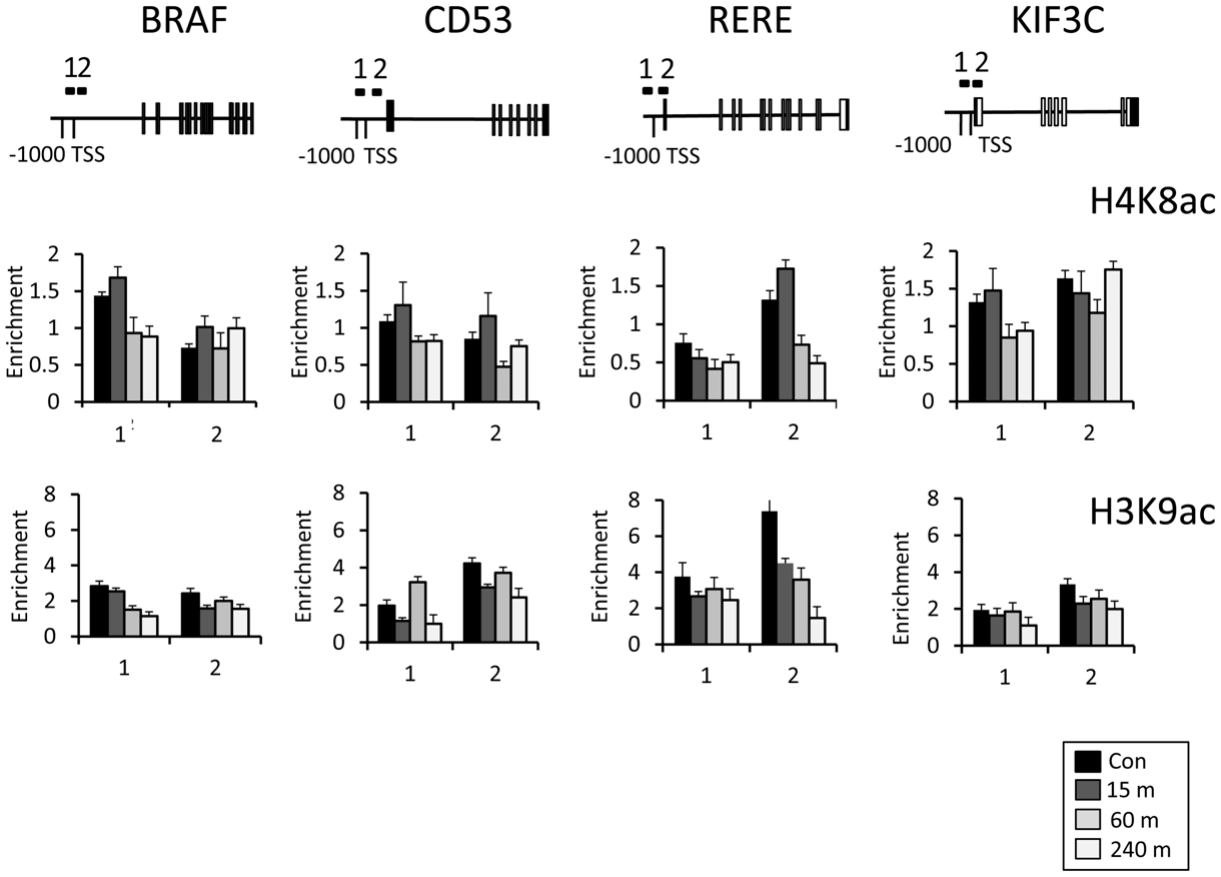
Histone acetylation changes on up-regulated genes during extended VPA treatment. Chromatin-immunoprecipitation analysis of H4K8ac and H3K9ac on up-regulated genes (*BRAF, CD53, RERE, KIF3C*) in response to VPA treatment (5 mM). Analysis was performed on either untreated cells **(Control)**, or during VPA treatment **(15, 60 & 240 minutes)**. Primer locations are indicated on the gene diagrams. The abundance of a histone modification (‘*Enrichment*’) is calculated from the ratio of material detected in bound∶unbound fractions.

The absence of significant increases in histone acetylation at VPA-induced genes was unexpected, but could reflect a gene's varied transcriptional response over the 8-hour treatment, which is not captured at a single timepoint. We therefore analysed histone acetylation at multiple points during the treatment, focussing on genes which are up-regulated over this timecourse (*BRAF, CD53, RERE, KIF3C;* Fig. 2B). We analysed acetyl marks (H3K9ac, H4K8ac) which have been extensively characterised, and shown to be associated with transcriptionally active promoters [13], However, as before, the abundances of these acetylated isoforms either remain unchanged, or show gradual and progressive decreases at all gene promoters (Fig. 4). Both marks tend to show similar behaviour at the same locus, but the decreases in H3K9ac are generally more marked (i.e. on the RERE promoter), reflecting its higher abundance in untreated cells.

**Figure 5:**
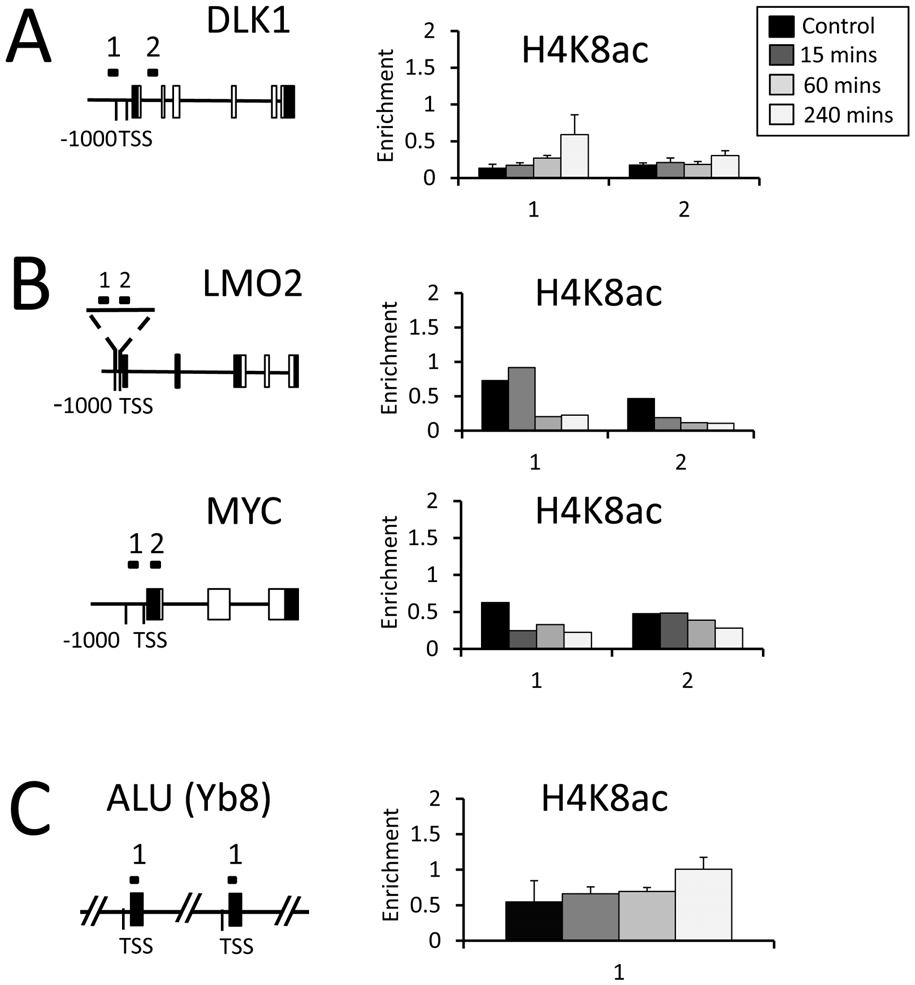
Histone acetylation changes on up- and down-regulated genes during extended VPA treatment. Chromatin-immunoprecipitation analysis of H4K8ac on: (**A**), Up-regulated gene (*DLK1*), (**B**), Down-regulated genes (*LMO2, MYC*) or (**C**), repetitive elements (*ALU*) in response to VPA treatment (5 mM). Analysis was performed on either untreated cells **(Control),** or during VPA treatment **(15, 60 & 240** **minutes).** Primer locations are indicated on the gene diagrams. The abundance of a histone modification (‘*Enrichment*’) is calculated from the ratio of material detected in bound∶unbound fractions.

Finally, we extended this experiment to analyse three classes of genes – (i) a gene those that showed early and progressive up-regulation (*DLK1,* Fig. 5A), (ii) genes that show down-regulation (*LMO2, MYC,* Fig. 5B), and (iii) an abundant repetitive element, ALU (Yb8) (Fig. 5C). In all cases H4K8ac abundance was low at promoter-proximal loci (i.e. B/UB rations lower than 1.0), however small progressive increases (i.e. *DLK1*) or decreases (i.e. *LMO2, MYC*) in the modification were detected though the time course. Interestingly, *ALU* repeat sequences, which account for ∼25% of the human genome, show a progressive increase in H4K8ac in VPA-treated cells, indicating that at least some repeat elements are susceptible to hyperacetylation in response to HDACi.

## Discussion

It has long been thought that the functional effects of HDACi are mediated by their ability to induce global histone hyperacetylation, which was presumed to induce transcriptional responses at key genes. However, recent studies indicate that large numbers of proteins are acetylated in response to HDACi, including key transcription factors and metabolic enzymes [16], suggesting that other mechanisms may contribute to HDACi activity. The data we present here suggest that these agents induce substantial changes in gene expression, but only at a subset of genes. Furthermore, the contribution of histone acetylation to this response is not straightforward.

Our results show that many genes are sheltered from the global histone hyperacetylation induced by HDACi. This is consistent with our finding that only a small proportion of genes show significant transcriptional changes in response to HDACi (*∼9%*), and that as many are down-regulated as activated, even after short periods of inhibitor treatment when transcriptional effects are likely to be direct (Fig. 2C). Furthermore, the finding that different inhibitors induce responses at distinct subsets of genes suggests that their functional effects are put in place via different pathways, rather than via their shared ability to induce histone hyperacetylation (Fig. 2A).

Subsequent analysis of histone modification distributions on VPA-responsive genes showed that, irrespective of transcriptional response, histone acetylation at gene promoters does not reflect the inhibitor–induced increase in bulk histone hyperacetylation. Even after longer inhibitor treatments, of the eight genes examined only one, DLK1, showed a modest increase in H4 acetylation. This is a small sample, but includes all the genes that show the largest transcriptional responses to HDACi treatment (Fig. 2B), suggesting that our findings are representative of most genes. It seems that local levels of histone acetylation are determined by gene specific factors rather than induced changes in global histone modification. While there are examples of individual promoters that fail to show enhanced acetylation in response to HDACi (e.g. MMTV, [26]), they have been seen as counterintuitive exceptions, and most reports focus on increased histone acetylation in response to HDACi (e.g., [13]). This remains controversial, as a recent study found HDACi induced transient increases in promoter acetylation at a subset of genes, but deacetylation after prolonged exposure (2–6 hrs) was a more typical response [14]. Our data is consistent with this, and suggests that many promoters show minimal change in response to HDACi and that genes showing increased acetylation, such as the *Hoxb* genes [27] are the exception rather than the rule.

## Supporting Information

Table S1 Primers used to assess changes in gene expression. Forward (F) and reverse (R) sequences are listed along with their melting temperatures (Tm). For some genes, more than one primer pair was used.
(DOC)

Table S2 Primers used for chromatin immunoprecipitation analysis. Forward (F) and reverse (R) primer sequences are listed along with melting temperatures (Tm).
(DOC)
