# Supplementary material for: Genes Are Often Sheltered from the Global Histone Hyperacetylation Induced by HDAC Inhibitors
Source: PLoS One. 2012 Mar 30;7(3):e33453. doi: 10.1371/journal.pone.0033453 (PMC3316569; doi:10.1371/journal.pone.0033453)
Supplement: Table S1 — Primers used to assess changes in gene expression. Forward (F) and reverse (R) sequences are listed along with their melting temperatures (Tm). For some genes, more than one primer pair was used. (DOC) [file pone.0033453.s001.doc]

**Supplementary Table S1**

| **Gene Name** |  | **Primer sequence** | **Tm**  **(^o^ C)** |
| --- | --- | --- | --- |
|  |  |  |  |
| *BRAF*: v-raf murine sarcoma viral oncogene homolog B1 | F | 5’-ACC ACC CAA TAC CAC AGG AA-3’ | 56 |
|  | R | 5’-CAT TGG GAG CTG ATG AGG AT-3’ | 54 |
| *CD53*: CD53 antigen | F | 5’-AGC ATC CAC CGT TAC CAC TC-3’ | 57 |
|  | R | 5’-ATG GTC TGG CTG GTT TTG TC-3’ | 56 |
| *KIF3C*: Kinesin family member 3C | F | 5’-TTG CCT GTT ACC CCT GTT TC-3’ | 55 |
|  | R | 5’-ATT CAC TCG CTT GCT TTC GT-3’ | 56 |
| *RERE*: arginine-glutamic acid dipeptide (RE) repeats | F | 5’-AGG ACA TAT GCC TGC CAA AG-3’ | 55 |
|  | R | 5’-CCA GGA TGA AGA GTG GGA CT-3’ | 56 |
| *HBEGF*: heparin-binding EGF-like growth factor | F | 5’-GGT GGT GCT GAA GCT CTT TC-3’ | 56 |
|  | R | 5’-CCC CTT GCC TTT CTT CTT TC-3’ | 54 |
| *VCL*: Vinculin | F | 5’-CTG TTC AAA CCA CTG AGG ATC A–3’ | 53 |
|  | R | 5’-TGC TGG TGG CAT ATC TCT CTT-3’ | 52 |
| *MYC*: v-myc myelocytomatosis viral related oncogene, | F | 5’-TTC GGG TAG TGG AAA ACC AG-3’ | 55 |
|  | R | 5’-CAG CAG CTC GAA TTT CTT CC-3’ | 55 |
| *LMO2*: LIM domain only 2 | F | 5’-AGG AAC CAG TGG ATG AGG TG-3’ | 56 |
|  | R | 5’-GAA TCC GCT TGT CAC AGG AT-3’ | 55 |
| *ACTB*: Actin, beta | F | 5’-TCC TGG GTA TGG AAT CTT GC-3’ | 54 |
|  | R | 5’-TGA GGC TAG CAT GAG GTG TG-3’ | 57 |
| *IL1B*: Interleukin 1, beta | F | 5' - GCTGAGGAAGATGCTGGTTC - 3' | 56 |
|  | R | 5' - CGTGCACATAAGCCTCGTTA - 3' | 55 |
|  | F | 5' - CAGCCAATCTTCATTGCTCA - 3' | 53 |
|  | R | 5' - GCATCTTCCTCAGCTTGTCC - 3' | 56 |
| *MCM7*: Minichromosome maintenance complex component 7 | F | 5' - TGCGCAGATTTGAGCTGTAT - 3' | 55 |
|  | R | 5' - CCACCATCTTGGGTTTGACT - 3' | 55 |
|  | F | 5' - GCCAAGTCTCAGCTCCTGTC - 3' | 57 |
|  | R | 5' - TCAGCACAGCTGCCGTAA - 3' | 57 |
| *PES1*: Pescadillo homolog 1, containing BRCT domain (zebrafish) | F | 5' - CAGGTTTCTCCTCCACGAAC - 3' | 55 |
|  | R | 5' - AGTGTTCCACTCGCTCTTCC - 3' | 57 |
|  | F | 5' - CGTTGGACTCCGAGAGTTGT - 3' | 57 |
|  | R | 5' - GTGGGAAACTCATCCACCTC - 3' | 55 |
| *DLK1*: Delta-like 1 homolog (Drosophila) | F | 5' - ATGCTGCGGAAGAAGAAGAA - 3' | 55 |
|  | R | 5' - TTGTCACACAGCAGCACAAA - 3' | 56 |
|  | F | 5' - ATGGATTCTGCGAGGATGAC - 3' | 55 |
|  | R | 5' - TCCACAGAGTCCGTGAAGG - 3' | 56 |
| *ANXA2*: Annexin A2 | F | 5' - GCCATCAAGACCAAAGGTGT - 3' | 55 |
|  | R | 5' - TCAGTGCTGATGCAAGTTCC - 3' | 56 |
| *MYC (c-Myc)* : v-myc myelocytomatosis viral oncogene homologue | F | 5' - GACTTGTTGCGGAAACGAC - 3' | 55 |
|  | R | 5' - GCACAAGAGTTCCGTAGCTG - 3' | 56 |
|  | F | 5' - GCGACTCTGAGGAGGAACAA - 3' | 56 |
|  | R | 5' - CCTGCCTCTTTTCCACAGAA - 3' | 55 |
|  | F | 5' - TTCGGGTAGTGGAAAACCAG - 3' | 54 |
|  | R | 5' - CACCGAGTCGTAGTCGAGGT - 3' | 58 |
