# Supplementary material for: Genes Are Often Sheltered from the Global Histone Hyperacetylation Induced by HDAC Inhibitors
Source: PLoS One. 2012 Mar 30;7(3):e33453. doi: 10.1371/journal.pone.0033453 (PMC3316569; doi:10.1371/journal.pone.0033453)
Supplement: Table S2 — Primers used for chromatin immunoprecipitation analysis. Forward (F) and reverse (R) primer sequences are listed along with melting temperatures (Tm). (DOC) [file pone.0033453.s002.doc]

**Supplementary Table S2**

| **Gene Name** | | **Primer Annotation** |  | **Primer sequence** | **Tm**  **(^o^ C)** | |
| --- | --- | --- | --- | --- | --- | --- |
|  |  |  |  |  |  |  |
| *KIF3C*: Kinesin family member 3C | | 2 | F | 5’-CGA GAG AAT CAG CTG GAA GG-3’ | 55 | |
|  |  |  | R | 5’-CTG TCC CGG TCT ACT TCT GG-3’ | 56 | |
|  |  | 1 | F | 5’-GCT AAG GGA AGG CTT CGT G-3’ | 56 | |
|  |  |  | R | 5’-ACT TAG TGC CCT GCC ACT TG-3’ | 57 | |
|  |  | 3 | F | 5’-CCC TGA TAG ACT CCG TGC TC-3’ | 57 | |
|  |  |  | R | 5’-TCC CCT GCA TGG TAT AGG TC-3’ | 55 | |
| *RERE*: Arginine-glutamic acid dipeptide (RE) repeats | | 1 | F | 5’-CCT CGG AAA ACA AAA CCA GA-3’ | 53 | |
|  |  |  | R | 5’-TTG AAC CTA GGC TCC CTC CT-3’ | 57 | |
|  |  | 2 | F | 5’-TGA CAG CGG ACA AAG ACA AA-3’ | 55 | |
|  |  |  | R | 5’-CGT GGC CTT GAA TTC TCA CT-3’ | 55 | |
|  |  | 3 | F | 5’-CGA CAG TGA GGA CAG TGA GC-3’ | 58 | |
|  |  |  | R | 5’-TAC TGG TGG TGA AGC AGT GG-3’ | 57 | |
|  |  | 1  (Fig 4) | F | 5’- ATC CGC GTC CTA ATG ACT GA – 3’ | 56 | |
|  |  |  | R | 5’ – CTT GCT GTT ACC CGG GTC TT - 3’ | 57 | |
|  |  | 2  (Fig 4) | F | 5’ – CCT CGG AAA ACA AAA CCA GA -3’ | 53 | |
|  |  |  | R | 5’ – TTG AAC CTA GGC TCC CTC CT – 3’ | 57 | |
| *LMO2*: LIM domain only 2 (rhombotin-like 1) | | 1 | F | 5' - CAG GCG TGT GTG TTG TCA G - 3' | 56 | |
|  |  |  | R | 5' - CCC ACA AGT CTC TTC CAA GC - 3' | 55 | |
|  |  | 2 | F | 5' - AGC AGA TTG CAA ACC AGG AC - 3' | 55 | |
|  |  |  | R | 5' - AGA CTC CGG CTG TCT TCT CTT - 3' | 57 | |
| ALU repeat: Yb8 | | 1 | F | 5' – CGA GGC GGG TGG ATC ATG AGG T - 3' | 63 | |
|  |  |  | R | 5' – TCT GTC GCC CAG GCC GGA CT - 3' | 65 | |
| *DLK1* : Delta-like 1 homologue (*Drosophila*) | | 1 | F | 5' – CTG GGT ATG TGT GCT TGT GG - 3' | 56 | |
|  |  |  | R | 5' – CAC AGC CAT GCA CCT GAT AC - 3' | 56 | |
|  |  | 2 | F | 5' – GAC GAC TTT GCC GTC TTA GC - 3' | 56 | |
|  |  |  | R | 5' – TCG CTG TCA GAG ACA GAT GG - 3' | 56 | |
| *MYC (c-Myc)* : v-myc myelocytomatosis viral oncogene homologue | | 1 | F | 5' – GAC TCA GTC TGG GTG GAA GG - 3' | 56 | |
|  |  |  | R | 5' – TGC ACA GCT ATC TGG ATT GG - 3' | 55 | |
|  |  | 2 | F | 5' – TAG GCG CGC GTA GTT AAT TC - 3' | 56 | |
|  |  |  | R | 5' – CAG CCG AGC ACT CTA GCT CT - 3' | 58 | |
| BRAF: v-raf  murine sarcoma  Viral oncogene  homologue B1 | | 1 | F | 5’– GAC CTC TTC TCT CCC AAT TGT TT – 3’ | 55 | |
|  |  |  | R | 5’- TCC AAG CAT TCT CTG CAA AA-3’ | 53 | |
|  |  | 2 | F | 5’- CTG CTT GGC TGT TTC AAC CT – 3’ | 56 | |
|  |  |  | R | 5’- TTT CCA CTG CAA TGA ACG TC - 3’ | 54 | |
|  |  | |  |  | |  |
| CD53: CD53 antigen | 1 | | F | 5’-TGG GTT GTC ACA TAC TCC TTT G – 3’ | | 55 |
|  |  |  | R | 5’ - CAC TAT GCT GCA TCA CTG GAA – 3’ | | 55 |
|  | 2 | | F | 5’- GAT TAT GCC TTG GGG ACT CA-3’ | | 54 |
|  |  |  | R | 5’- TTG AAT TGG ACA CCA TTT GC-3’ | | 52 |
|  |  | |  |  | | |
